# Supplementary material for: Orbital selective commensurate modulations of the local density of states in ScV6Sn6 probed by nuclear spins
Source: Nat Commun. 2024 Sep 18;15:8213. doi: 10.1038/s41467-024-52456-6 (PMC11411110; doi:10.1038/s41467-024-52456-6)
Supplement: Supplementary file 1 — Supplementary Information [file 41467_2024_52456_MOESM1_ESM.pdf]

SUPPLEMENTARY INFORMATION

Orbital selective commensurate modulations of the local density of states in ScV_6Sn_6 probed by nuclear spins

Robin Guehne,[1,*] Jonathan Noky,[1] Changjiang Yi,[1] Chandra Shekhar,[1] Maia G. Vergniory,[1,2] Michael Baenitz,[1] and Claudia Felser[1]

[1]*Max Planck Institute for Chemical Physics of Solids, 01187, Dresden, Germany*

[2]*Donostia International Physics Center, 20018 Donostia - San Sebastian, Spain*

*robin.guehne@cpfs.mpg.de

The following supplement comprises additional experimental data that constitutes essential complementary evidence to the already documented results which did not make it into the main file for reason of space. We will first present temperature dependent spectra for the $c \parallel B_0$ orientation, detailed orientation dependent NMR spectra that allows us to evaluate the electric field gradient (EFG) at the V nuclei for high and low temperatures. We will next show how the NMR shift is extracted for the $a^* \parallel B_0$ spectrum where the central transitions are hidden and provide values of the NMR shift anisotropy. This is followed by the in-plane patterns of the orbital specific DOS. Next, we show evidence that the total signal intensity can be brought into consistent agreement with changes in temperature, circuit performance, and rf-penetration depth. We will further provide some field dependent measurements, as well as a detailed analysis of the high temperature spin-lattice relaxation to conclude on its magnetic origin.

Supplementary Note 1: Temperature dependent ^{51}V spectra for $c \parallel B_0$

In Supplementary Fig. 1 we show a detailed account of the spectral changes for temperatures between 170 and 20 K and $c \parallel B_0$. The highlighted range of temperatures denotes the CDW phase transition where the high temperature spectrum is progressively replaced by the 3 low temperature spectra.

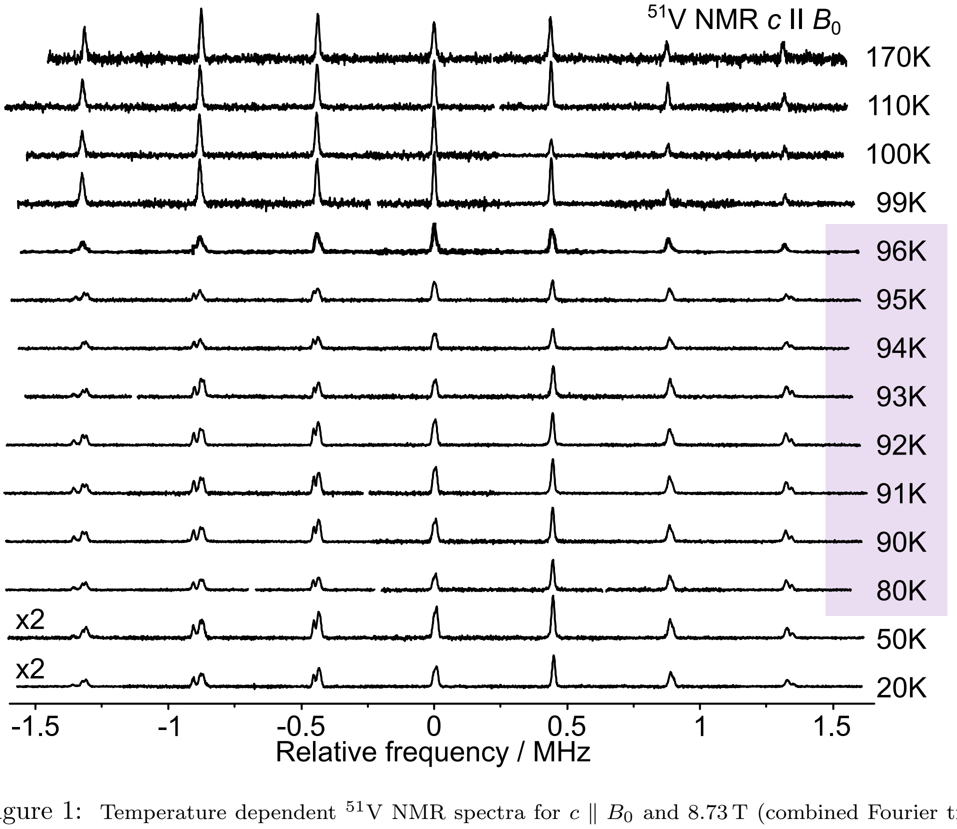

Supplementary Figure 1: Temperature dependent ^{51}V NMR spectra for $c \parallel B_0$ and 8.73 T (combined Fourier transformed selective spin echoes). The individual noise levels reflect different signal averaging. The spectra were corrected for the temperature, signal averaging, and for the rf-circuit's quality factor. The two lowermost spectra (50 and 20 K) are further rescaled by ×2 for clarity. The highlighted temperature range marks the phase transition. The peculiar low temperature intensity pattern, especially the pronounced first high frequency satellite, reveal changes in the NMR shift in addition to the obvious differences in the quadrupole splitting frequencies.

Supplementary Note 2: Evaluation of the EFG tensor at high and low temperatures

In the following Supplementary Figs. 2 to 5 the results of detailed orientation dependent measurements are provided. The experiments were carried out for 170 K (Supplementary Figs. 2 to 4) and 80 K (Supplementary Fig. 5) at a magnetic field at 8.73 T using broad band FIDs (0.5 μs) and stepwise rotation of the single crystal placed on a single axis goniometer. When rotating the EFG about one of its principle axis (X, Y or Z) the apparent quadrupole splitting $\tilde{\nu}_Q$ of the observed spectrum changes according to

$$\tilde{\nu}_Q = \frac{\nu_Q}{2}(3\cos^2\beta - 1 + \eta \sin^2\beta \cos 2\alpha) \qquad (1)$$

where ν_Q denotes the quadrupole splitting frequency defined in the main text and α and β are the Euler angles in the PAS of the EFGs.

Note, the various orientation dependent measurements are carried out to consistently determine the orientation of the EFG with respect to the lattice. Size (V_{ZZ}) and shape (η) are obtained from measurements with the single crystal being well adjusted along the Z and Y direction and making use of the EFG being traceless and the definition of the asymmetry parameter.

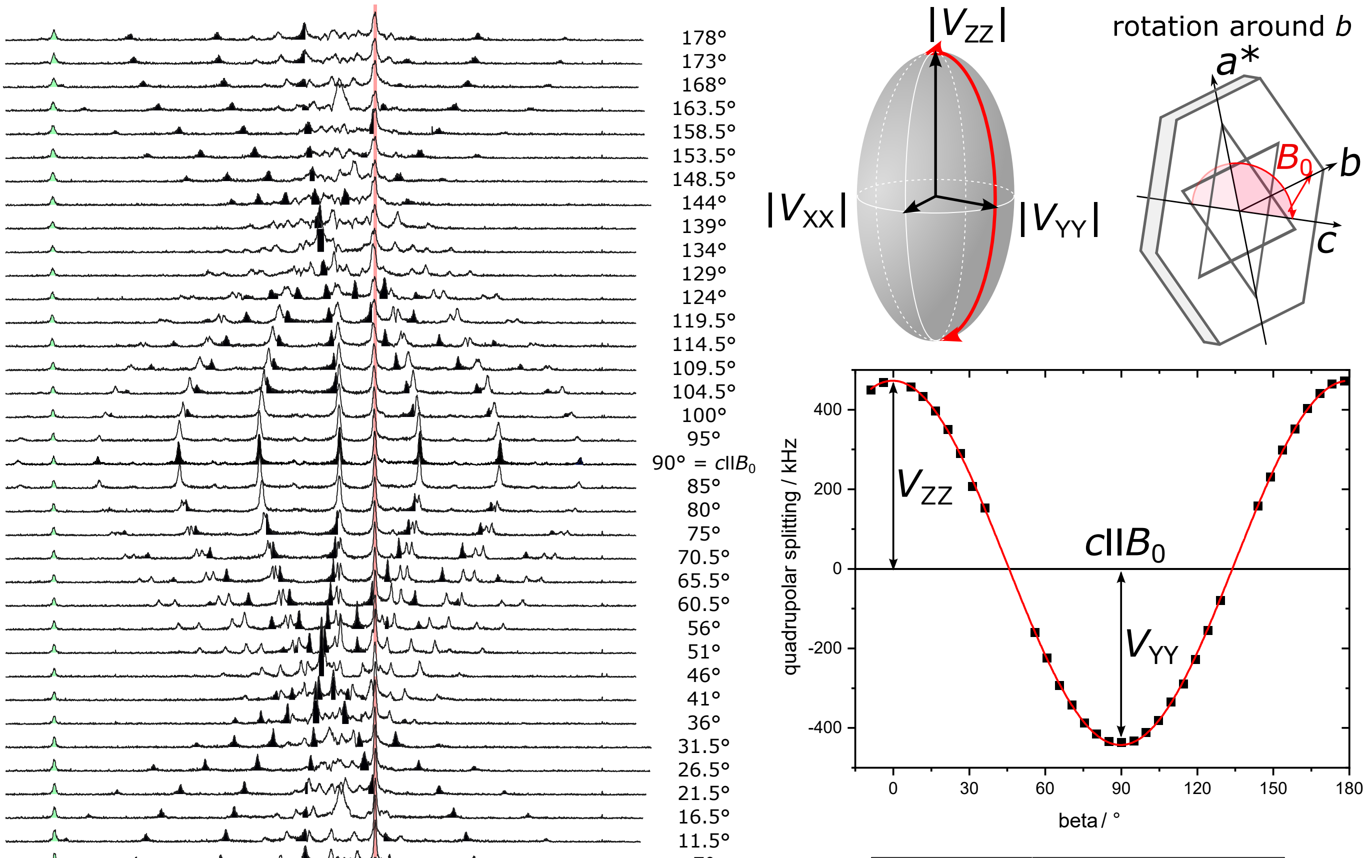

vQ	472.9059±1.29522
d	-0.24983 ± 0.07866
eta	0.87239 ± 0.00663
alpha	90 ± 0

Supplementary Figure 2: Orientation dependent broad band NMR spectra at 170 K and 8.73 T for crystal rotations around the crystal b-axis which corresponds to a rotation about the X-axis of the EFG's PAS (gray ellipsoid). Thus, the rotation connects V_{ZZ} and V_{YY}. Note, only the black resonances belong to this well defined rotation, while unmarked resonances represent the other two V nuclei per V triangle that undergo a rotation that leaves the PAS and can thus only be evaluated with much more difficulty. In addition, just above 97 MHz and at about 98.75 MHz the isotropic ^{27}Al (light green) and ^{63}Cu (light red) resonance lines, respectively, can be seen. The signal stem from a small piece of Al metal inside, and from the Cu wire of the rf-coil. The plot on the right hand side shows the quadrupole splitting as function of angle, including the typical fit using the formula above and keeping $\alpha = 90°$.

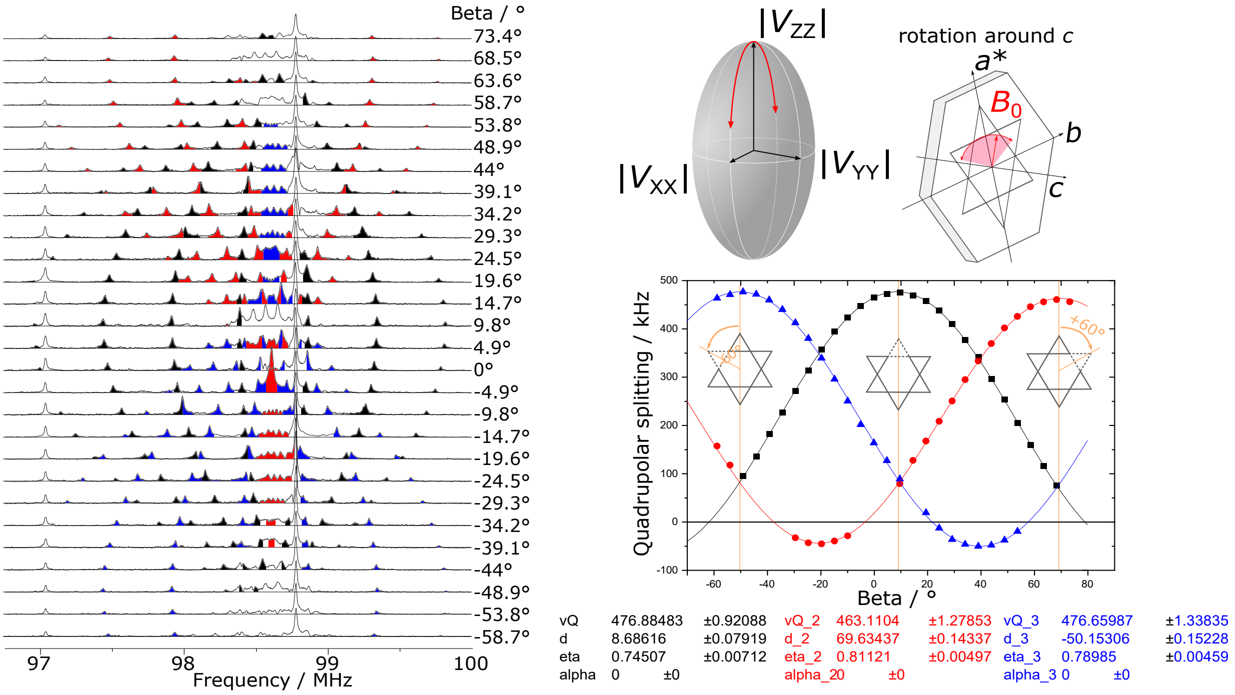

vQ	476.88483	±0.92088	vQ_2	463.1104	±1.27853	vQ_3	476.65987	±1.33835
d	8.68616	±0.07919	d_2	69.63437	±0.14337	d_3	-50.15306	±0.15228
eta	0.74507	±0.00712	eta_2	0.81121	±0.00497	eta_3	0.78985	±0.00459
alpha	0	±0	alpha_20		±0	alpha_3 0		±0

Supplementary Figure 3: Orientation dependent broad band NMR spectra at 170 K and 8.73 T for crystal rotations around the crystal c-axis which corresponds to a rotation about the Y-axis of the EFG's PAS (gray ellipsoid). Thus, the rotation connects V_{ZZ} and V_{XX}, the values of which are easily extracted. Note, since the PAS of the EFG's of each of the three V nuclei per V triangle share the Z-axis, all of the resonances (black, blue, and red) can be used to evaluate the EFGs as shown in the plot on the right hand side. Again, the isotropic ^{27}Al and ^{63}Cu resonance lines are visible. This time, $\alpha = 0°$ and the phase shift $d \approx \pm 60°$ determines the relative orientation of the three EFG's and the sixfold symmetry of the Kagome lattice.

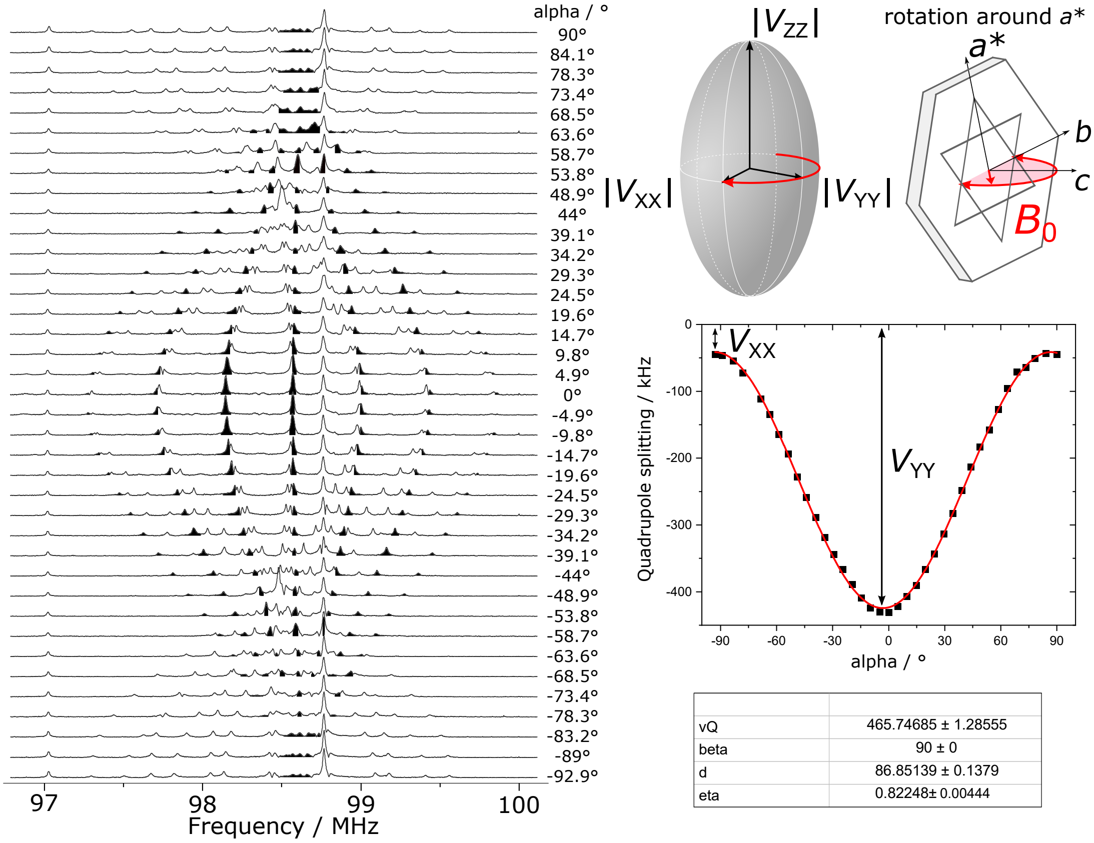

vQ	465.74685 ± 1.28555
beta	90 ± 0
d	86.85139 ± 0.1379
eta	0.82248± 0.00444

Supplementary Figure 4: Orientation dependent broad band NMR spectra at 170 K and 8.73 T for crystal rotations around the crystal a^*-axis which corresponds to a rotation about the Z-axis of the EFG's PAS (gray ellipsoid). Thus, the rotation connects V_{YY} and V_{XX}. Note, only the black resonances belong to this well defined rotation, while unmarked resonances belong to the other two V nuclei per V triangle that undergo a rotation that leaves their PAS and can thus only be evaluated with much more difficulty. The plot on the right hand side shows the quadrupole splitting as function of angle, including the typical fit using the formula above while keeping $\beta = 90°$.

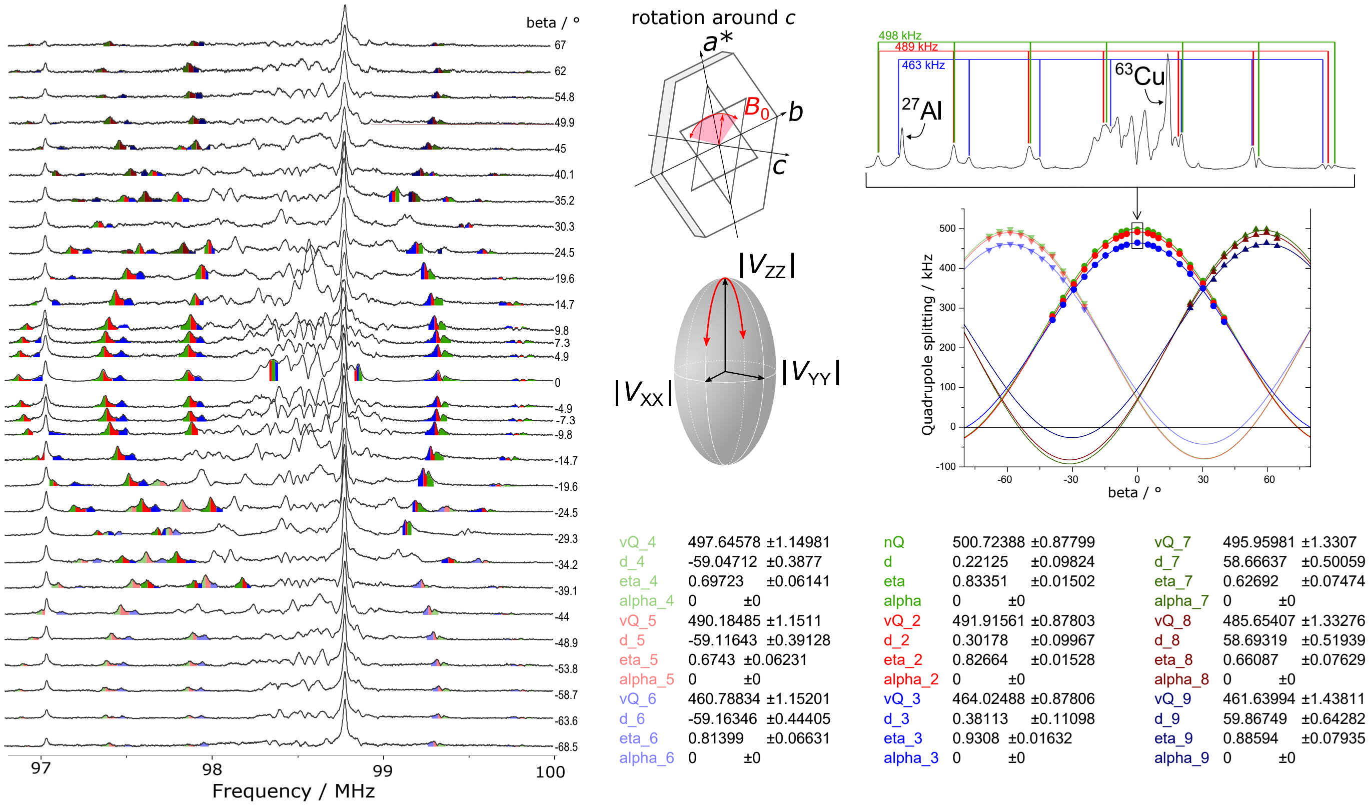

Supplementary Figure 5: Orientation dependent broad band NMR spectra at 80 K and 8.73 T for crystal rotations around the crystal c-axis similar to the one shown in Supplementary Fig. 3. In the CDW phase, the NMR spectrum visible splits into 3 individual spectra for each of the 3 V per V triangle (green, red, and blue – in light, regular, and dark color) while the sixfold symmetry of the Kagome lattice is retained. The plot on the right documents the orientation dependence and sixfold symmetry of the underlaying lattice.

Supplementary Note 3: Determination of the NMR shift for the in-plane orientation

For $c \parallel B_0$ the central transitions and thus the NMR shift are directly accessible as can be seen in Fig. 4 (**a**) in the main file. For the $a^* \parallel B_0$ orientation, the CTs (for high and low temperatures) are hidden behind the broad quadrupole spectrum of the 2 other V nuclei in each V triangle. In order to obtain the NMR shift, therefore, the satellites can be used, and the first order (equidistant) quadrupole pattern. The procedure is shown in Supplementary Fig. 6. The relevant low temperature spectrum consists of 3 equally intense quadrupole patterns differing in shift and quadrupole splitting (purple, orange, and light blue).

Supplementary Note 4: NMR shift anisotropy

For the high temperatures, the NMR shift was found to be axially symmetric, as there is essentially no change in the resonance frequency of the CT in Supplementary Fig. 4. It is a reasonable assumption that the CDW phase inherits this symmetry. The corresponding isotropic ($K_{\text{iso}} = (2K_c + K_{a^*})/3$) and axial ($K_{\text{axial}} = 2(K_c - K_{a^*})/3$) shift components are shown in Supplementary Fig. 7. The colors follow Fig. 4 (**c**) and (**d**) of the main manuscript and are identified with the high single temperature resonance V and three chemically non-equivalent low temperature V sites V1, V2, and V3. In the CDW phase, the isotropic shifts assume 3 different values, from about 0.743 % for V1 to about 0.76 % for V3. Surprisingly, their mean value (gray column in panel **c**) agrees almost exactly with K_{iso} for the high temperature resonance just above the onset of CDW phase transition at 96 K (black column). Contrastingly, the anisotropy of all 3 low temperature V sites increases, yielding values between −0.14 % (V3) to almost −0.17 % (V2). The mean axial shift component (gray) deviates clearly from the high temperature K_{axial} at 96 K, with a difference in the order of 190 ppm (0.019 %). Thus, one way to look at the CDW transition is through the shift anisotropy, with a net change in the axial part of the shift (ΔK_{axial}), while the average isotropic shift remains unaltered in comparison to the high temperature phase.

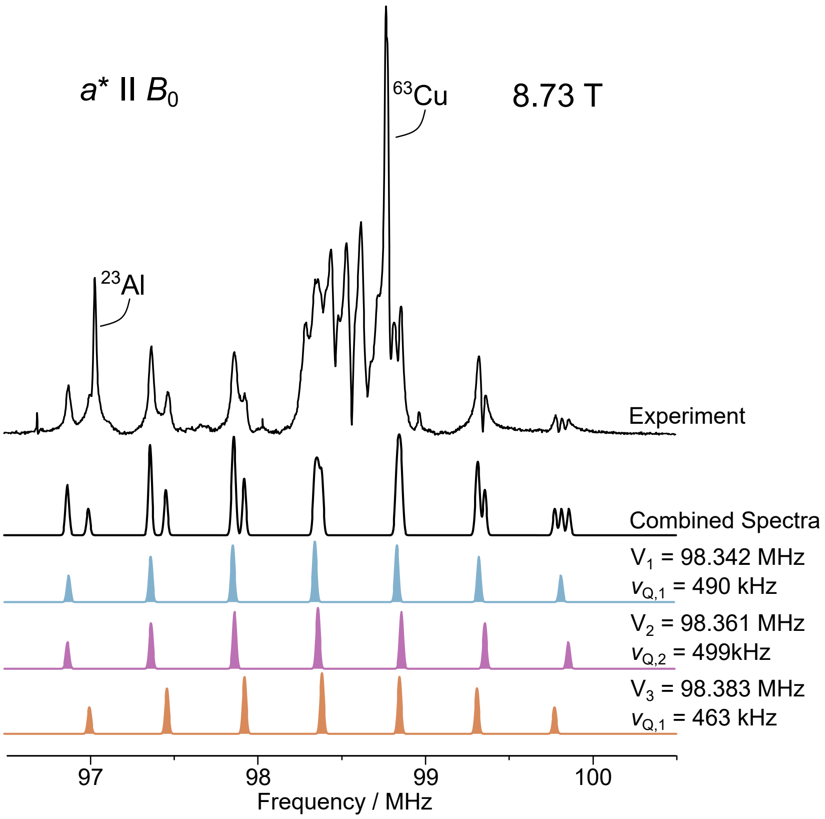

Supplementary Figure 6: Fourier transform of a broad band NMR signal for 80 K and 8.73 T with the single crystal aligned as $a^* \parallel B_0$. The four spectra below represent the three individual quadrupole patterns (colored) and their sum (black).

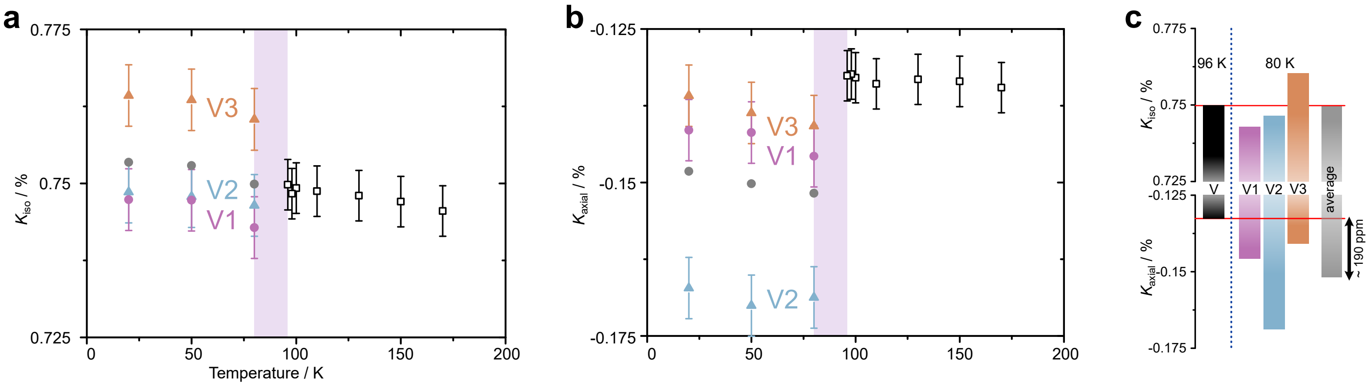

Supplementary Figure 7: (**a**) The isotropic and (**b**) axial shift components as functions of temperature. Error bars denote the uncertainty to determine individual resonance frequencies due to finite line broadening. Note that for low temperatures an axial symmetry cannot be verified with the experiment. The values during the phase transition are missing because for $a^* \parallel B_0$ it is not possible to identify individual resonances due to the overlap of resonances. (**c**) the isotropic and axial shift components for 96 and 80 K, as well as the low temperature average (gray columns). As can be seen, the average isotropic shift does not change during the charge density wave phase transition, while the anisotropic or axial component changes by about 190 ppm.

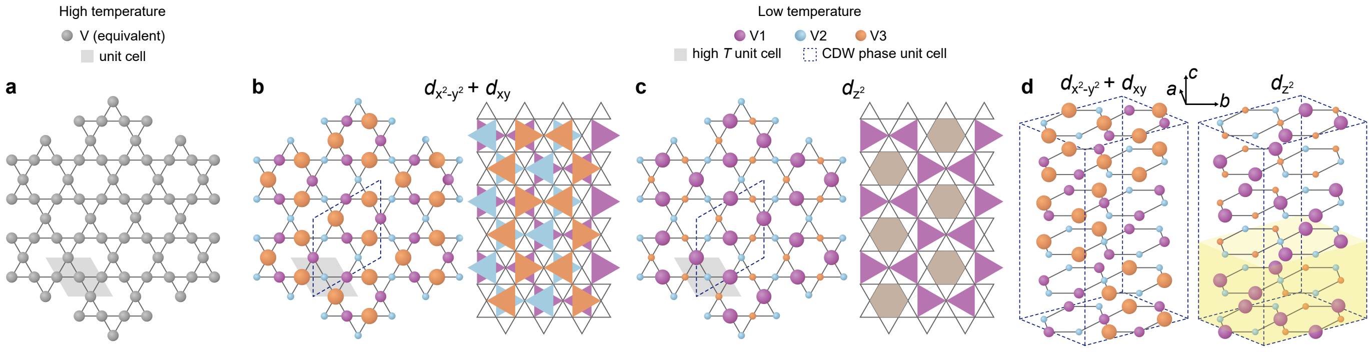

Supplementary Figure 8: (**a**) At high temperatures, the local DOS is evenly distributed among the equivalent V atoms (gray circles). The unit cell is represented as the gray rhombus. At low temperatures in the CDW phase, the local DOS is modulated. (**b**) For the equivalent in-plane orbitals, $d_{x^2-y^2} + d_{xy}$, this modulation is three-fold, i.e., each chemically non-equivalent crystal site (color) has a different DOS (size of the ball). In comparison to the high temperature phase, the unit cell is enlarged and rotated (dashed rhombus). Connecting crystal sites of equal DOS yields the colored patterns on the right, consisting of enlarged and intersecting Kagome networks (similar patterns are obtained for the equivalent $d_{xz} + d_{yz}$ orbitals and for the EFGs). (**c**) For the out-of-plane orbitals d_{z^2}, V2 and V3 (blue and orange) have the same DOS (ball size) which allows them to be connected in closed hexagons centered in the enlarged Kagome network of V1. The corresponding unit cell is the same as in (**b**). (**d**) The complete unit cell of the two DOS modulations including the stacking along c. Again, since the DOS of V2 and V3 in case of the out-of-plane orbitals d_{z^2} are equal, the wavelength of the modulation along c contains only 3 kagome layers (yellow box) instead of six as in the case of the in-plane orbitals $d_{x^2-y^2} + d_{xy}$, constituting $\mathbf{q}_{z^2} = (\frac{1}{3}, \frac{1}{3}, \frac{2}{3})$.

Supplementary Note 5: Kagome plane patterns of orbital selective DOS

The NMR shift splittings observed for the two orientations $c \parallel B_0$ and $a^* \parallel B_0$ reflect the local magnetic field to be modulated in the crystal structure. DFT calculations suggest that the origin is a modulation of the site and orbital selective DOS as discussed in Fig. 6 of the main manuscript. When regarding the Kagome plane alone, different DOS patterns emerge for the different V d orbitals. In Supplementary Fig. 8 we show these patterns in comparison with the unmodulated case. As reported by [1], the unit cell grows and rotates with the emergence of the CDW (gray and dashed rhombuses). When connecting sites of equal DOS, two distinct patterns appear for the two considered orbitals, $d_{xz} + d_{yz}$ and d_{z^2}. That is, orbital selective DOS forms a substructure in the CDW which may be of importance in the context of transport phenomena in future studies.

Supplementary Note 6: The circuit's quality factor, rf-penetration depth, and the total signal intensity

To evaluate the total signal intensity we investigate the central transition of Fig. 4 (**a**) in the main file. This allows us to eliminate some uncertainties that are connected with the excitation conditions in terms of selective excitations, power levels, band widths etc. The CTs are nevertheless representative for the total signal intensity. We reproduce the spectra in Supplementary Fig. 9 (**a**). These spectra are corrected for signal averaging as well as for the temperature. An NMR signal is further proportional to $\sqrt{Q}$, where Q is the quality factor of the rf-circuit. We plot Q as a function of temperature in panel (**b**). Q changes with temperature since it is connected to the resistivity of the sample. We thus corrected the signal intensity given by the gray area under the curves in panel (**a**) by the changes in Q, and plot it in panel (**c**) as gray stars. Finally, since the resistivity of the sample changes significantly as a function of temperature [2], we estimated the corresponding penetration depth of the rf-field at $f = 100\,\mathrm{MHz}$ due to the skin effect, using $\delta(T) = \sqrt{2\rho(T)/(2\pi f \mu_0)}$, where ρ denotes the sample resistivity, f the frequency, and μ_0 the vacuum magnetic permeability. The results are plotted as purple triangles in panel (**c**). Consistently, as the resistivity decreases with decreasing temperature, the skin depth decreases as well, thus, reducing the number of nuclear accessible with NMR. Obviously, the total signal intensity's dependence on temperature follows very well the changes of the skin depth expected for a thick sample ($200\,\mu$m). This analysis confirms that we have no unusual intensity loss in the present system.

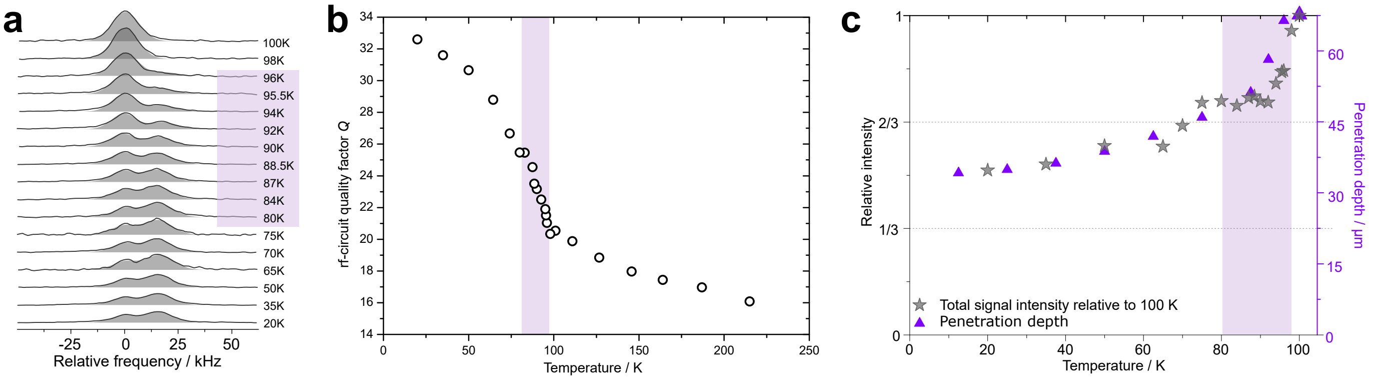

Supplementary Figure 9: (**a**) The CT for $c \parallel B_0$ as a function of temperature reproduced from Fig. 4 in the main manuscript file. The gray areas correspond to the total signal intensity. (**b**) The rf-circuit's quality factor Q as a function of temperature. Clearly, a bend in the dependence is visible at the onset of the CDW phase transition, most potentially due to the corresponding change in the sample resistivity and the thus changing rf-penetration depth that affects the inductance of the rf-coil. (**c**) The CT signal intensity from panel (**a**) after correction for the temperature and Q fits matches almost perfectly with the T dependence of the rf-penetration depth (rescaled to match the 100 K value of the intensity) implying the loss in signal intensity is due to a loss in accessible nuclei from a reduced penetration depth.

Supplementary Note 7: Magnetic field dependent measurements

The double-peak central transition structure from Fig. 4 (**a**) in the main manuscript was measured for two more magnetic fields. The spectra are shown in Supplementary Fig. 10 in units of ppm. Evidently, the two peaks do not change in relative position (150 ppm), nor in relative intensity. This is a strong evidence that the origin of this peak system is related to different Knight shift values, i.e., different DOS as expected from a CDW. Second order quadrupole effects ($\propto 1/B_0$) obviously do not affect the spectrum, while, on the other hand, unusual magnetism as potentially related to orbital currents, are not evident.

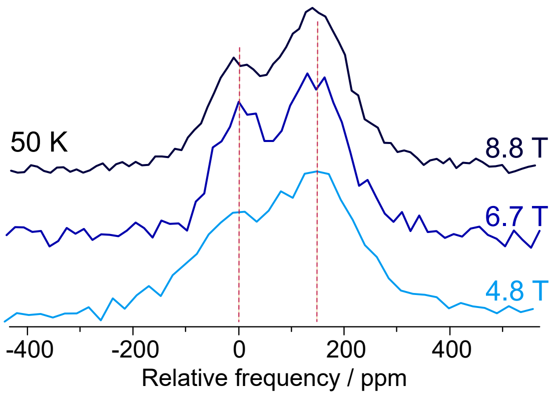

Supplementary Figure 10: Fourier transform of spin-echo measurements of the central double-peak for $c \parallel B_0$ and 50 K for external fields of 4.8, 6.7, and 8.8 T in units of ppm. The red dashed lines denote the approximate center frequencies of the two peaks. A constant splitting in units of ppm proves the origin of the splitting is due to different NMR shifts (paramagnetic spin susceptibility) rather then an unusual magnetism.

Supplementary Note 8: Magnetic relaxation and determination of T_1

In a quadrupolar split system, it can be difficult to determine T_1, as it is defined to be the relaxation time of the unsplit spin system, i.e., as if no quadrupole interaction is present. For ^{51}V, the quadrupole spectrum covers almost 3 MHz and thus, the relaxation of individual transitions is the only way to measure T_1 for fairly well defined conditions. The relaxation of individual transition, however, depends on the transition and the relaxation mechanism, i.e., whether its driven by magnetic fluctuations due to free carriers or lattice vibrations via the quadrupole interaction.

In order to check the relaxation mechanism in the current system, we assume the relaxation to be of magnetic origin, and apply the corresponding recovery equations to extract T_1. We used selective saturation recovery pulse sequences and analyzed the individual recovery curves with:

$$M(t) = M_0 \left[1 - f \left\{ \sum_{i=1}^{7} a_i \cdot \exp\left(-\frac{\lambda_i t}{T_1} \right) \right\} \right] , \tag{2}$$

where M_0 is the equilibrium signal intensity, and f the inversion factor ($f = 1$ saturation, $f = 2$ inversion). The coefficients a_i vary for each transition, while the exponents λ_i are shared. We summarize the various values in Supplementary Tab. 1.

Supplementary Table 1: Coefficients a_i and exponents λ_i for magnetic relaxation and recovery measurements of selectively saturated or inverted transitions of a spin 7/2 system.

a_i	CT	1st Sat	2nd Sat	3rd Sat	λ_1
$i = 1$	1/84	1/84	1/84	1/84	1
2	0	1/84	1/21	3/21	3
3	3/44	1/33	1/132	3/11	6
4	0	9/77	25/308	25/77	10
5	75/364	1/1092	100/273	75/364	15
6	0	49/132	49/132	3/44	21
7	1225/1716	392/858	98/858	8/858	28

The results are shown in Supplementary Fig. 11. Black denotes the recovery for the CT, red for the first, petrol for the second, and pink for the third lower satellite. The results provide strong evidence for the assumption, because each of the 4 transition selective recoveries, i.e, CT and the three satellites, yield the same T_1 of 24(1) ms at 250 K and $c \parallel B_0$. We further repeated the measurement for the 1st satellite at the same temperature for $a^* \parallel B_0$ (blue data), which yields the same result, and hence, the relaxation is isotropic.

Finally, there are a few crystal orientations where one of the 3 V quadrupole splittings disappears, and therefore, the quadrupole interaction is eliminated. We used $\angle(c, B_0) \approx 75^\circ$ (cf. Supplementary Fig. 2) to measure the pure T_1 of the un-split spin system (Supplementary Fig. 11 yellow data) and applied the simple exponential relationship

$$M(t) = M_0 \left[1 - f \cdot \exp\left(-\frac{t}{T_1} \right) \right] \tag{3}$$

which, evidently, gives the same T_1 as for selective excitation in the other orientations. We have thus confirmed the relaxation to be driven by magnetic fluctuations from free electrons which is the prerequisite for our treatment of $1/T_1(T)$ using the DOS.

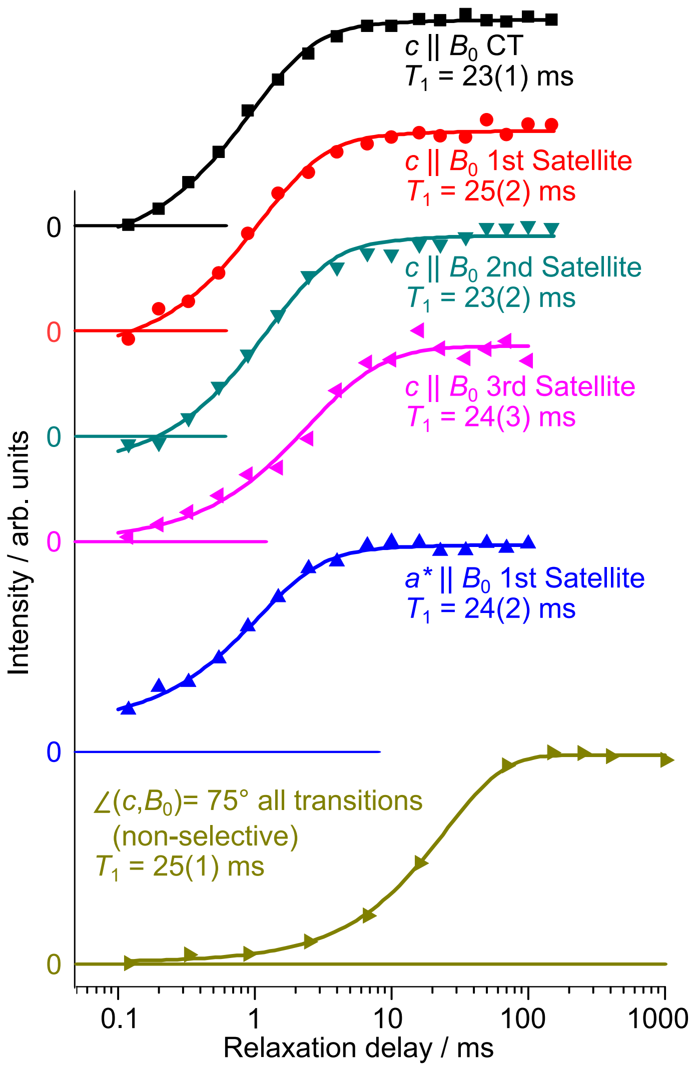

Supplementary Figure 11: Evaluation of the spin-lattice relaxation at 250 K and 8.73 T. The plot shows the results from individual saturation recovery measurements carried out for the CT (black), and the first (red), second (petrol), and third (pink) satellite of the V spectrum for $c \parallel B_0$, as well as for the first satellite (blue) for $a^* \parallel B_0$, and for the non-selective spectrum at 75° (yellow). The solid curves denote the best fit using equations (2), (3), and Supplementary Tab. 1.

Supplementary References

[1] Hasitha W. Suriya Arachchige, William R. Meier, Madalynn Marshall, Takahiro Matsuoka, Rui Xue, Michael A. McGuire, Raphael P. Hermann, Huibo Cao, and David Mandrus. Charge Density Wave in Kagome Lattice Intermetallic ScV_6Sn_6. *Phys. Rev. Lett.*, 129:216402, Nov 2022. doi: 10.1103/PhysRevLett.129.216402.

[2] Changjiang Yi, Xiaolong Feng, Ning Mao, Premakumar Yanda, Subhajit Roychowdhury, Yang Zhang, Claudia Felser, and Chandra Shekhar. Quantum oscillations revealing topological band in kagome metal ScV_6Sn_6. *Phys. Rev. B*, 109:035124, Jan 2024. doi: 10.1103/PhysRevB.109.035124.
